# Knowledge and associated factors of obstetric fistula among antenatal care attendees at Faji Kunda and Farafenni health facilities, The Gambia

**DOI:** 10.1371/journal.pone.0331130

**Published:** 2025-09-24

**Authors:** Amadou Bah, Gbolahan Oladele Obajimi, Baboucar Cham, Musa Jaiteh

**Affiliations:** 1 Reproductive Health Sciences Program, Pan African University Life and Earth Sciences Institute (Including Health and Agriculture), University of Ibadan, Ibadan, Nigeria; 2 Department of Obstetrics and Gynecology, College of Medicine, University of Ibadan, Ibadan, Nigeria; 3 Gambia College School of Nursing and Midwifery, Banjul, The Gambia; 4 South African Medical Research Council/University of Johannesburg (SAMRC/UJ) Pan African Centre for Epidemics Research (PACER) Extramural Unit, Faculty of Health Sciences, University of Johannesburg, Johannesburg, South Africa; Injibara University, ETHIOPIA

## Abstract

An obstetric fistula is an inappropriate communication between the vagina and surrounding tubular organs, usually, the rectum and bladder, resulting in leakage. The World Health Organisation estimates that 50,000–100,000 women worldwide suffer from obstetric fistula each year. In-country literature reveals Gambian women know little about obstetric fistula. No published data existed on antennal women’s perspectives and obstetric fistula knowledge factors. This study examines obstetric fistula knowledge and associated factors among antenatal care attendants at Faji Kunda and Farafenni Health Facilities, The Gambia. A facility analytical cross-sectional study included 385 antenatal care attendees. Study participants were recruited using systematic sampling. Statistical Package for the Social Science (SPSS) version 24 cleaned and stored the data, while Statistical Software Package (SATA) version 18 analysed it. Tables with percentages showed descriptive data. Parallel multivariate logistic regression and Chi-square were used to determine obstetric fistula knowledge factors. The significance level was p < 0.05 with a 95% CI. Most study participants (37.92%) were 18–23 years old, and 37.4% (n = 144) had no formal schooling. The study found that 24.2% (n = 93) of participants knew adequately about obstetric fistula. Results show a significant association (p < 0.001) between obstetric fistula knowledge and age. Results indicate a strong association (p < 0.001) between obstetric fistula knowledge and education levels. Higher chances are observed for tertiary education attendees [aOR=8.55; p < 0.001; 95% CI (2.83–25.81)]. Awareness of obstetric fistula was substantially linked to occupation, particularly among government personnel [aOR=18.6; p < 0.001; 95% CI (5.29–65.38)]. A higher connection was found among attendees from wealthy backgrounds [aOR=7.41; p < 0.001; 95% CI (2.40–22.84)]. The study participants knew little about obstetric fistulas. According to the study, age, education, income index, and occupation were significantly associated with obstetric fistula knowledge. Therefore, a widespread campaign to promote girls’ education and obstetric fistula awareness is needed.

## Introduction

Obstetric fistula occurs when there is abnormal communication between the vagina and nearby tubular structures, typically the bladder, rectum or both, which results in leakage. [1] Obstetric fistula continues to be primarily disregarded despite the profound consequences it has on women, families, and society at large [2]. Globally, obstetric fistula has a yearly incidence of 50,000–100,000 women [3]. Ghana, in particular, documented that Obstetric fistula affects 1,352 women per 751,205 births annually, with an incidence rate of 1.8 per 1000 deliveries [4]. An estimated 2.6 per cent of women in Uganda of reproductive age (about 142,000 women) have experienced obstetric fistula; in Central and Eastern Uganda, that percentage rises to 2.8 per cent [5]. In The Gambia, the frequency of fistula remains uncertain; nevertheless, estimations drawn from maternal and neonatal death rates, treatment statistics, and contextual information place the number of cases between 0.46 and 2.05 per 1000 women [6]. Obstetric fistula is mainly caused by delayed/prolonged and obstructed labor, without timely access to high-quality obstetric care [7]. This delay leads to ischemic necrosis tissue and resulting to abnormal opening between the vagina and the bladder (vesicovaginal fistula) or rectum (rectovaginal fistula) [8]. In low-income countries, several factors, like shortage of trained birth attendants, poor health-seeking behaviors, few facilities offering comprehensive obstetric care services, malnutrition, poverty, illiteracy, underage marriage and childbirth, harmful traditional practices, sexual violence, and a lack of timely maternal and health care are significant contributors of obstetric fistula particularly in underdeveloped regions where adequate obstetric care is lacking [7,9]. In contrast, in high-income countries, iatrogenic injuries during cesarean sections or hysterectomies are now the leading cause of obstetric fistula [10].

This issue continues to be overlooked, particularly in middle-income nations with limited resources, and it poses substantial health concerns to women as a result of socioeconomic obstacles; The Gambia is no exception. [11]. A significant proportion of those impacted are young women who reside in socioeconomically disadvantaged rural regions. These regions are marked by inadequate healthcare services, low socioeconomic standing, and educational obstacles, all of which impede their ability to obtain appropriate treatment [12]. A study in rural Tanzania reveals that women could not promptly access health facilities that provided comprehensive emergency obstetric care due to a lack of financial resources, decision-making authority, and transportation access. Furthermore, those women who managed to reach the facilities received substandard birth care [13].

A study in Ethiopia identified six distinct attributes that impact women’s comprehension of obstetric fistula. These attributes comprise educational attainment, occupation, receipt of obstetric fistula counselling, attendance at pregnancy-related conferences, antenatal care follow-up visits, residency status, and possession of television or radio [14].

Constant difficulties such as incontinence, social isolation, shame, and a variety of health problems plague those who are impacted. Over 2 million young women in Asia and Sub-Saharan Africa were estimated to have untreated obstetric fistulas in 2018. [3]. Nonetheless, according to research conducted in India, the frequency of obstetric fistulas was inadequate (0.08 per cent). However, due to the community-based nature of the study, the results could not be generalized. [15]. In The Gambia, the frequency of fistula remains uncertain; nevertheless, estimations drawn from maternal and neonatal death rates, treatment statistics, and contextual information place the number of cases between 0.46 and 2.05 per 1000 women. [6]. Obstetric fistula awareness was found to be limited among reproductive-age women in The Gambia, according to research. [12]. This study aimed to determine the level of knowledge and associated factors of obstetric fistula among antenatal attendees at Faji Kunda and Farafenni Health Facilities, The Gambia.

## Methodology

### Study area and period

The study was conducted at Farafenni Reproductive Maternal Newborn Child and Adolescent Health (FRMNCAH) and Faji Kunda Major Health Center. Farafenni RMNCAH is a rural facility located in Farafenni, North Bank Region; it is 170 km inland from the capital, Banjul. The facility offers antenatal care services, adolescent services, and infant and child health services. Faji Kunda Health Center is located in the urban area of The Gambia. Faji Kunda Major Health Center is the sole major hospital in Western Region 1. The facility has an outreach population of about 300,000. The facility has a labor ward, a pediatric ward, and a laboratory. The data was collected from May 5 to July 3, 2023.

### Study design

An analytical cross-sectional study was conducted among antenatal care attendees at the Farafenni Reproductive Maternal Newborn Child and Adolescent Health (FRMNCAH) and Faji Kunda Major Health Centre to evaluate their knowledge and associated factors about obstetric fistula.

### Study Population

Antenatal women visiting the Farafenni (FRMNCAH) and Faji Kunda Major Health Center clinics. The estimated number of antenatal women attending these facilities during data collection was 1210.

### Inclusion criteria

All Antenatal care attendees registered with Farafenni RMNCAH and Faji Kunda Major Health Center were included in the research and accepted to participate.

### Exclusion criteria

Antenatal women who are not mentally sound, and those who are under the age of eighteen years, because of the requirement for consent. Those who were deaf/dumb were also excluded because it was not easy to get a sign-language translator.

### Sample size determination

This study recruited 385 antenatal care attendees using the Kish and Leslie (1965) formula.


$$n_{\text{0}}=\;\frac{Zx^{\text{2}}PQ}{d²}=\frac{(\text{1}.\text{96}{)}^{\text{2}}\;\times \text{0}.\text{5 }\times \text{0}.\text{5}}{\text{0}.\text{05}²}=\text{385}$$

- Where $n_{0}$ (sample size) = 385
- d = precision = 0.05
- p = 50%
- Z = 95% CI
- Q = 1-p

Based on the antenatal population sizes of Faji Kunda and Farafenni, the sample size was divided between them using the formula n1 = (n X N1)/ N, which was adapted from (Shakil, 2019).

Where:

- n = the total sample size = 385
- N1 = total population for group 1 (Faji Kunda) = 858
- N_2_ = the total population for group 2 (Farafenni) = 352
- N = the total population in the two groups = 1210
- n1 = population of Faji Kunda (270)
- n2 = n-n1 = 385−270 = 115 (population of Farafenni)

### Sampling technique

Antenatal attendees registered with the facilities participating in this study were recruited using a systematic sampling technique, and the data collection period for this study spanned two months, with one month allocated for participant selection in each facility. The participants were identified using the antenatal attendees’ list as a sampling frame. The urban health facility conducted twelve antenatal visits, while the rural health facility conducted eight antenatal visits. The sampling interval (x) was determined for each antenatal clinic visit by dividing the number of eligible antenatal women (N) by the number of attendees intended to be selected for that visit (n). A random ballot was used to determine the initial participant (Y), and subsequent participants were chosen using Y + x, Y + 2x, etc. If an eligible antenatal attendee declines to participate or provides consent for an interview, the following participant on the list is chosen.

The participants were duly informed of the study’s aims and advantages, and their informed consent was obtained. Individual interviews were conducted with all consenting antenatal women, during which information regarding their sociodemographic characteristics and understanding of obstetric fistula was gathered. Adequately trained research assistants collected the data.

### Data collection procedure

Structured questionnaires conducted by interviewers were used to gather data. The questionnaire was developed using pertinent data from other studies. [7,16]. The study objectives created two sections of the questionnaire. The information on sociodemographic variables (age, residence, occupation, educational level, marital status, and wealth index) was presented in the first section. The residence of the respondents was grouped into rural and urban. The wealth was constructed into (poorest, poor, medium, rich, and richest) using Principal Component Analysis (PCA), following the methodologies outlined by Filmer and Pritchett (2001) and Rutstein and Johnson (2004) [17]. The participants’ knowledge was assessed using a nine-item scale that categorized their understanding as poor or good. The questions encompassed various aspects of obstetric fistula, including its causes, risk factors, preventive measures, and treatment options. Every accurate response was assigned a score of 1, while any incorrect or uncertain response was assigned a score of 0. The responses were divided into two percentage categories: 0–49% (poor) and 50–100%. (good).

An association test was used to identify the factors related to knowledge of obstetric fistulas by comparing independent variables to the outcome variable.

### Validity and reliability

The questionnaires were distributed to experts, including two professors, for the content validity test, and changes were made in response to their suggestions. Furthermore, thirty antenatal women conveniently selected from different locations pretested the questionnaires. Using the same respondents, the test-retest method was applied with a two-week gap between the two tests. This made it easier to make the required changes to the questionnaire and gave us a way to assess how suitable the questions were to guarantee validity and accurate answers. Assessing the responses’ consistency was also beneficial. The reliability of the questionnaire was evaluated using the Pearson correlation test, and the overall reliability score was 0.83 (r = 0.83).

### Study variables

There are independent and dependent variables in the study.

**Dependent variable**: Knowledge of obstetric fistula.

**Independent variables**: Socio-demographic characteristics (age, gender, marital status, residence, level of education, wealth index, occupation, number of pregnancies, parity, and age of first marriage).

### Data collection methods

Six second-year nursing students were hired as research assistants to gather data. This is because their training was more straightforward, and they were already familiar with research. Before beginning data collection, the principal investigator trained the research assistants for two days. The structured questionnaire was utilized to collect data under the supervision of a research assistant. The questions were made available in three of the most widely spoken local languages in The Gambia: “Wollof,” “Mandinka,” and “Fula.” These languages are generally simple to comprehend throughout The Gambia. Two people were asked to translate the questionnaire back into English to ensure it was translated correctly. Individual interviews took place in an office setting, ensuring participant privacy at all times. The participants were advised before the session that the interview would last approximately thirty minutes and that the data gathered would be kept private. The researcher or assistants collected data in the language the participants understood best. Two months were spent assembling the data.

### Data analysis

After each administration, the researcher and assistants verified the questionnaires in the study area to ensure completeness. Participants were identified using codes to enhance data entry into the Statistical Package for the Social Sciences (SPSS) version 24, and the data were cleaned and appropriately coded. Verifying missing data or incorrect data entry was done before the data analysis, and there was no missing data. The data was analyzed using the Statistical Software Package (STATA) software version 18. Frequencies and percentages were utilized to tabulate the variables. In the analysis, both descriptive and inferential statistics were employed. The significance of the association between the independent and dependent variables was examined using the Pearson Chi-square test. A multiple logistic model was constructed to further show which independent variables were significantly associated with knowledge about obstetric fistula. The multiple logistic regression analysis was conducted with a significance level of P < 0.05.

### Ethical consideration

Ethical approval was obtained from The University of Ibadan/University College Hospital Ethical Committee (UI/UCH EC-UI/EC/23/0063) of the Institutional Review Board, Regional Health Directorate, Western 1 Region and Regional Health Directorate, North Bank East Region. The participants also provided their informed consent. Participants were selected for the study after showing that they were ready to participate by signing the Informed Consent form. Before they could sign or thumbprint the consent form, they were instructed to understand the study in straightforward terms fully. The participants in the study incurred no financial costs. The public health field’s adherence to autonomy, confidentiality, privacy, beneficence, non-maleficence, and justice was guaranteed.

### Inclusivity in global research

Additional information regarding the ethical, cultural, and scientific considerations specific to inclusivity in global research is included in the Supporting Information (SX Checklist).

## Results

### Participants’ socio-demographic characteristics

The study included all 385 participants selected from various study locations in its analysis (Table 1).

**Table 1:** Participants’ socio-demographic characteristics of participants (n = 385).

Variable	Rural	Urban
	N (%)	N (%)
**Age in years**		
18-23	70 (18.2)	76 (19.7)
24-29	21 (5.5)	105 (27.3)
>29	24 (6.3)	89 (23.3)
**Marital status**		
Single/divorced	3 (0.8)	4 (1.1)
Married	112 (29.1)	266 (69.1)
**Educational level**		
No formal	68 (17.7)	76 (19.7)
Primary	22 (5.7)	61 (15.8)
Secondary	18 (4.7)	92 (23.9)
Tertiary	7 (1.8)	41 (10.6)
**Occupational status**		
Private/business/others	22 (5.7)	60 (14.6)
Housewife	85 (22.1)	176 (45.7)
Government	8 (2.1)	34 (8.8)
**Residence**	115 (29.9)	270 (70.1)
**Wealth index**		
Poor	50 (13.0)	45 (11.7)
Medium	49 (12.7)	165 (42.9)
Rich	16 (4.2)	60 (15.6)
**Husband educational level**		
No formal	83 (21.6)	91 (23.6)
Primary	4 (1.0)	21 (5.5)
Secondary	17 (4.4)	62 (16.1)
Tertiary	11 (2.9)	96 (24.9)
**Husband occupational status**		
Government	13 (3.4)	52 (13.5)
Private	8 (2.1)	47 (12.2)
Business	33 (8.6)	110 (28.6)
Others	61 (15.9	51 (15.8)

The majority of the study participants reside in the urban areas, accounting for 70.1% (n = 270) of the total sample, compared to 29.9% (n = 115) in rural areas. Age distribution indicated that urban participants were generally older, with 105 (23.7%) aged 24–29 years and 89 (23.3%) aged 29 years and older, compared to 21 (5.5%) and 24 (6.3%) respectively in rural areas. Almost all women were married, 112 (29.1% rural, 266 (69.1% urban). Educational attainment indicated that more urban participants, 41 (10.6%), attained tertiary education compared to 7 (1.8%) of rural women. A similar trend was also observed in terms of employment, where more women in the urban setting are employed in the government (34; 8.8%), private and business (60; 14.6%), and rural (8; 2.1%), private and business (22; 5.7%). Being a housewife was common in both settings. A clear socio-economic division was observed between the two settings: rural women were more in the poor wealth category (50; 13.0%) compared to urban women (45; 11.7%), whereas urban participants were predominantly in the medium (165; 42.9%) and rich (60; 15.6%) categories.

In terms of husbands’/partners’ educational level and employment, educational attainment in urban areas was higher (96; 24.9%) compared to rural (11; 2.%). Moreover, urban husbands were more often employed in government (52;13.5%), private (47; 12.2%), or business (110; 28.6%) sectors, while rural husbands were more concentrated in other informal occupations (61; 15.9%).

### Participants’ obstetric history

Obstetric characteristics revealed that teenage pregnancy (15–19 years) was common in both rural and urban areas, but slightly more frequent in urban participants (135;35.1%) than in rural participants (71; 18.4%) (Table 2). First pregnancies at the age of 20–24 years were also notable, particularly among urban women (100; 26.0% versus 38; 9.9% in rural areas). Parity patterns indicated that rural women had more high-parity pregnancies, more than four, with 37 (9.6%) compared to 90 (23.4%) in urban areas, while 1–3 pregnancies were more common in urban settings (180; 46.8%) than rural settings (78; 20.3%). Birth complications were more reported among urban women (55; 14.3%) compared to rural women (26; 6.8%). Most participants across both settings had between 0 and 3 children (89; 23.1%, rural, 227; 59.0% urban), although rural women who had more than three children (26.8%) were more than their urban counterparts (11.2%).

**Table 2:** Participants’ obstetric characteristics (n = 385).

Variable	Rural N (%)	Urban N (%)
**Age at first pregnancy**		
15-19	71 (18.4)	135 (35.1)
20-24	38 (9.9)	100 (26.0)
>24	6 (1.6)	35 (9.1)
**Number of pregnancies**		
1-3	78 (20.3)	180 (46.8)
>4	37 (9.6)	90 (23.4)
**Developed birth complications**		
Yes	26 (6.8)	55 (14.3)
No	89 (23.1)	215 (55.8)
**Number of children**		
0-3	89 (23.1)	227 (59.0)
>3	26 (6.8)	43 (11.2)

### Participants’ level of knowledge

The respondents’ knowledge level was categorized into good and poor; participants who scored 50–100% were classified as good, and those who scored 0–49% were categorized as poor (Table 3). This categorization was adapted from Azanu et al. (2020) [18].

**Table 3:** Parameters and scoring system used to assess knowledge of obstetric fistula.

Score Range	Knowledge Level	Description
50-100%	Good knowledge	Participants correctly answered at least half of the knowledge questions.
0-49%	Poor knowledge	Participants answered less than half correctly.

**Fig 1:**
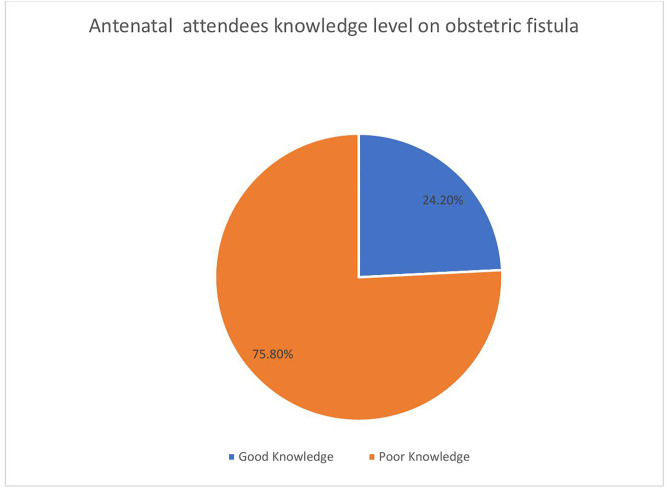
Antenatal attendees’ knowledge level on obstetric fistula.

The results (Fig 1) show that 75.8% (n = 292) of the antenatal attendees displayed good knowledge regarding obstetric fistula. In comparison, only 24.2% (n = 93) of the participants demonstrated good knowledge about obstetric fistula.

**Fig 2:**
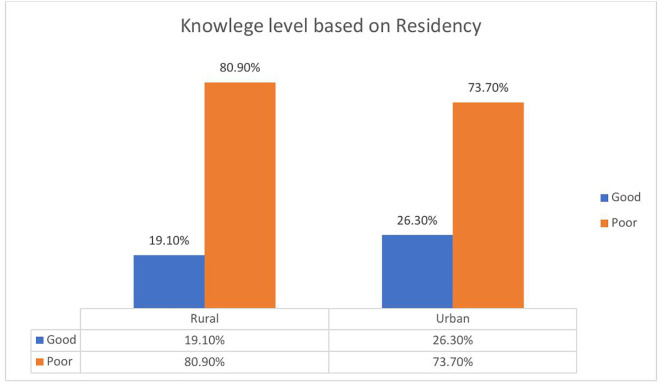
Antenatal attendees’ Knowledge level based on residency.

The chart illustrates that most antenatal attendees, whether from rural or urban areas, exhibit poor knowledge regarding obstetric fistula (19.1% and 26.3%, respectively). Urban attendees show a slightly higher percentage (26.3%) of knowledge than their rural counterparts (Fig. 2).

### Association between Socio-demographic characteristics and knowledge about obstetric fistula

The results from the chi-square analysis revealed that there is a significant association between knowledge about obstetric fistula and the following variables: age (p-0.001), participants’ educational level (p < 0.001), occupational status (P < 0.001), wealth index (p < 0.001), husband/partners educational level (p-0.021) and husband occupational status (p-0.042) (Table 4). The other variables show no significant association with knowledge about obstetric fistula.

**Table 4:** Chi-Square Analysis Showing Factors Predicting Women’s Level of Knowledge on Obstetric Fistula (n = 385).

Variables	Knowledge			X^2^	p-value
	Good n(%)	Poor n(%)	Total		
**Age**				**14.3903**	0.001*
18-23	22(15.1)	124(84.9)	146		
24-29	31(24.6)	95(75.4)	126		
>29	40(35.4)	73(64.6)	113		
**Marital status**				**2.2707**	**0.132**
Single	0(0)	7(100)	7		
Married	93(24.6)	285(75.4)	378		
**Educational level**				**51.0093**	0.000*
No formal	23(16.0)	121(84.0)	144		
Primary	10(12.1)	73(87.9)	83		
Secondary	30(27.3)	80(72.7)	110		
Tertiary	30(62.5)	18((62.5)	48		
**Occupational status**				**67.5130**	0.000*
Private	8(9.8)	74(90.2)	82		
Housewife	54(20.7)	207(79.3)	261		
Government	31(73.8)	11(26.2)	42		
**Residence**				**2.2604**	**0.133**
Urban	71(26.3)	199(73.7)	270		
Rural	22(19.1)	93(80.9)	115		
**Wealth index**				**51.1653**	0.000*
Poor	8(8.4)	87(91.6)	95		
Medium	44(20.6)	170(79.4)	214		
Rich	41(53.9)	35(46.1)	76		
**Husband educational level**				**9.7123**	0.021*
No formal	30(17.2)	144(82.8)	174		
Primary	8(32.0)	17(68.0)	25		
Secondary	20(25.3)	59(74.7)	79		
Tertiary	35(32.7)	72(67.3)	107		
**Husband occupational status**				**8.2012**	0.042*
Government	24(36.9)	41(63.1)	65		
Private	15(27.3)	40(72.7)	55		
Business	30(21.0)	113(79.0)	143		
others	24(19.7)	98(80.3)	122		

(*) = significant p-value (p < 0.05).

According to the findings presented in (Table 5), there was not a significant association between the variables about obstetric features and the level of knowledge of obstetric fistula.

**Table 5:** Chi-square analysis between obstetric characteristics and knowledge about obstetric fistula (n = 385).

Variables	Knowledge			X^2^	p-value
	Good n(%)	Poor n(%)	Total		
**Age of first pregnancy**				**5.1281**	**0.077**
15-19	41(19.9)	165(80.1)	206		
20-24	38(27.5)	100(72.5)	138		
>24	14(34.1)	27(65.9)	41		
**Number of pregnancies**				**2.5634**	**0.109**
1-3	56(21.7)	202(78.3)	258		
>3	37(29.1)	90(70.9)	127		
**Number of children**				**1.0703**	**0.3011**
0-3	73(23.1)	243(76.9)	316		
>3	20(29.0)	49(71.0)	69		

(*) = significant p-value (p < 0.05).

Table 6 depicts the outcomes of multivariate logistic regression, examining how sociodemographic factors relate to antenatal attendees’ knowledge about obstetric fistula. The findings underscore a notable and statistically significant association between age and knowledge of obstetric fistula. Aged 24–29 was significantly associated with knowledge about obstetric fistula [p-0.028; 95% CI (0.10–0.89)]. Furthermore, the results highlight a significant association between educational attainment and knowledge of obstetric fistula. Attendees with tertiary education exhibit notably higher odds [aOR=8.55; p < 0.001; 95% CI (2.83–25.81)] of having a good knowledge of obstetric fistula compared to those with no formal education. Additionally, secondary school graduates have a 2.23 times greater odds ratio to possess knowledge about obstetric fistulas. Moreover, this association has a confidence interval of 95% (0.99–5.05), meaning that they are 2.23 times more likely to know about obstetric fistula than those with no educational attainment.

**Table 6:** Multiple Logistics Regression Showing Factors Predicting Women’s Level of Knowledge on Obstetric Fistula (n = 385).

Factors	Knowledge			aOR	p-value	95% CI
	Good n(%)	Poor n(%)	Total			
**Age**						
18-23	22(15.1)	124(84.9)	146	Ref		
24-29	31(24.6)	95(75.4)	126	0.29	0.028*	0.10-0.89
>29	40(35.4)	73(64.6)	113	0.41	0.059	0.16-1.04
**Educational level**						
No formal	23(16.0)	121(84.0)	144	Ref		
Primary	10(12.1)	73(87.9)	83	0.60	0.311	0.22-1.62
Secondary	30(27.3)	80(72.7)	110	2.23	0.056	0.99-5.05
Tertiary	30(62.5)	18((62.5)	48	8.55	0.000*	2.83-25.81
**Occupational status**						
Private	8(9.8)	74(90.2)	82	Ref		
Housewife	54(20.7)	207(79.3)	261	3.62	0.006*	1.44-9.04
Government	31(73.8)	11(26.2)	42	18.59	0.000*	5.29-65.38
**Wealth index**						
Poor	8(8.4)	87(91.6)	95	Ref		
Medium	44(20.6)	170(79.4)	214	1.99	0.164	0.76-5.22
Rich	41(53.9)	35(46.1)	76	7.41	0.000*	2.40-22.84
**Husband educational level**						
No formal	30(17.2)	144(82.8)	174	Ref		
Primary	8(32.0)	17(68.0)	25	5.96	0.004*	1.76-20.16
Secondary	20(25.3)	59(74.7)	79	1.30	0.540	0.56-3.01
Tertiary	35(32.7)	72(67.3)	107	0.48	0.143	0.18-1.28
**Husband occupational status**						
Government	24(36.9)	41(63.1)	65	Ref		
Private	15(27.3)	40(72.7)	55	0.39	0.073	0.14-1.09
Business	30(21.0)	113(79.0)	143	0.75	0.555	0.29-1.96
others	24(19.7)	98(80.3)	122	0.75	0.604	0.26-2.19

(*) = significant p-value (p < 0.05) aOR = Odds Ratio CI = Confidence Interval.

The analysis also revealed compelling associations between certain factors and the knowledge about obstetric fistula. Occupationally, government personnel showed a substantial likelihood of being knowledgeable about the condition, with an adjusted odds ratio [(aOR=18.6; p < 0.001; 95% CI (5.29–65.38)], indicating they were 18.6 times more likely to possess this knowledge. Likewise, attendees from wealthier backgrounds exhibited a stronger association, with those in the rich category having an [aOR=7.41; p < 0.001; 95% CI (2.40–22.84)], indicating they were 7.4 times more likely to have comprehensive knowledge about fistula. Moreover, those with moderate wealth were twice as likely to have such knowledge. Additionally, the educational level and occupation of the husband/partner were significantly related to obstetric fistula knowledge (p = 0.021 and p = 0.042, respectively).

## Discussion

The research revealed that most of the pregnant women involved (37.7%) were between 18 and 23 years old. This finding aligns with a study conducted in Bangladesh, where a large portion (68.5%) of participants were between 16 and 25 years old, with an average age of 23.24 [19]. This finding might be associated with The Gambia’s median age of first marriage, reported as 19 years based on Gambian DHS 2019−20 data. The research emphasizes a concerning pattern in the levels of knowledge exhibited by antenatal attendees: a mere 24.2 per cent demonstrated a good knowledge of obstetric fistula. This discovery is consistent with prior research in Burkina Faso and Ethiopia, which documented 36% and 36.4% low awareness rates, respectively [7,14]. The limited understanding of obstetric fistula may be attributed to a lack of discourse during prenatal and antenatal visits. Furthermore, the lack of formal education among most pregnant women in this research probably impeded their comprehension of the condition. Likewise, non-native English speakers may find interpreting material on obstetric fistula challenging because most media dialogues are in English, creating a language barrier. This knowledge gap highlights the criticality of implementing health education strategies that are both linguistically accessible and culturally sensitive during antenatal care. Such an approach would increase understanding and awareness of obstetric fistula among diverse populations.

However, an examination of fourteen Sub-Saharan African nations revealed that the percentage of knowledge among women in Uganda was significantly higher, at 63.9% [20]. The differences in knowledge level might be due to different methodologies and sample sizes. The above study used secondary data extracted from demographic health survey data sets.

The research findings indicated a marginal disparity in the levels of knowledge held by antenatal attendants in rural and urban areas; urban participants demonstrated a slightly higher knowledge (26.3 per cent) than their rural counterparts (19.3 per cent). This discovery is consistent with findings from other studies. For example, [21] a lower proportion of rural inhabitants were found than their urban counterparts who possessed sufficient preventative information regarding obstetric fistula. Another research conducted in Ethiopia revealed that the odds of knowing obstetric fistula were approximately two times higher among respondents who resided in urban than those who resided in rural [9].

These differences in knowledge between urban and rural highlight the importance of tailoring awareness efforts to meet the unique needs of the rural communities, ensuring better sensitization and prevention through targeted information and outreach programs.

The research findings highlight a significant statistical association between age and understanding of obstetric fistula. In particular, there is a lower chance of being aware of obstetric fistula among pregnant women aged 24–29 years and above 29 years, as shown by adjusted odds ratios (aOR) of 0.45 and 0.29, respectively, in comparison to younger persons. Notably, these results are consistent with research conducted by [20], which examined fourteen Sub-Saharan African (SSA) nations. Age was also recognized as a critical factor related to obstetric fistula awareness in the study by [20]. The consistent findings seen in several research provide further support for the notion that age is a significant factor affecting knowledge of obstetric fistula.

The results of this research emphasize a significant association between educational attainment and knowledge of obstetric fistula. Compared to those with no formal education, individuals with tertiary education had considerably greater chances (aOR=8.55; p < 0.001; 95% CI (2.83–25.81)) of having a comprehensive understanding of obstetric fistula. Furthermore, these results are consistent with several Ethiopian research that found a significant association between women’s education level and their ability to be aware of obstetric fistula [1,9,14]. Additionally, an identical pattern was identified in research carried out in Bangladesh [19]. It was shown that women without secondary school education were 85 per cent less likely to have good awareness than those with tertiary education [aOR = 0.162; 95% CI (0.081–0.364)]. Similar results in these research studies emphasize the critical significance of education in influencing women’s awareness levels concerning obstetric fistula. According to the present study’s findings, there is a significant association between occupation and understanding of obstetric fistula. In particular, those whom the government employed had a significantly greater probability of possessing knowledge regarding obstetric fistula, as shown by an adjusted odds ratio (aOR) of 18.6 (p < 0.001; 95 per cent CI (5.29–65.38). This finding indicates that their likelihood of possessing knowledge regarding the disease was 18.6 times greater than that of homemakers. This conclusion correlates with research done in Ghana by [16], which similarly found employment, parity, and educational background as critical characteristics of knowledge about obstetric fistula. Moreover, a comparable and consistent trend is observed in an additional investigation carried out by [14], which disclosed that various factors, including residence, occupation, education level, obstetric fistula counseling, attendance at conferences for pregnant women, ANC follow-up, place of employment, and access to mass media were predictive elements associated with women’s understanding of obstetric fistula. These results reinforce the significance of work and other sociodemographic variables in shaping the degree of knowledge among women regarding obstetric fistulas.

A significant association was observed between the socioeconomic status of the participants and their level of understanding regarding obstetric fistula. In particular, those categorized as rich showed a considerable probability, as seen by an adjusted odds ratio (aOR) of 7.41 (p < 0.001; 95 per cent CI (2.40–22.84)), suggesting that they had a 7.4-fold increased likelihood of possessing extensive information about fistula. Furthermore, individuals classified as moderately wealthy exhibited a twofold increase in the probability of possessing such information compared to those classified as poor. The results of this research are consistent with those of [20], who examined sub-Saharan African nations and found a significant correlation between knowledge of obstetric fistulas and the wealth index. The study’s findings indicated a decreased probability of women in lower wealth index groups having information regarding obstetric fistula than women in higher wealth index groups. The aforementioned continuous trend underscores the significant impact that socioeconomic class and affluence have on the degree of knowledge that women possess regarding obstetric fistula.

According to the results of this study, obstetric fistula knowledge is not significantly associated with parity, number of pregnancies and the age of the first pregnancy. In contrast, the results of alternative research suggested a distinct association between parity and women’s knowledge of obstetric fistula. According to that study, women have a lower degree of understanding regarding obstetric fistulas when parity declines [20]. The opposing outcomes underscore a divergence between the current study’s findings concerning knowledge about obstetric fistula and parity. This implies that there may be some variation in how various reproductive circumstances influence knowledge levels of obstetric fistula among women; hence, the necessity for more research and comprehension of these intricate interactions is highlighted.

## Limitations of the study

The drawback of this study is that its results may not be fully generalized to all women because it was facility-based. Due to the legal age of consent in The Gambia, we could not include antenatal attendees under 18, who are essential in an obstetric fistula study.

## Implications of the study

According to the study, women generally know very little about obstetric fistula. Public health professionals should start a widespread awareness campaign, with a focus on rural areas, to counteract this low level of knowledge about the condition. If women were informed about the causes, symptoms, and treatments of the condition, we could have a nation with zero obstetric fistulas.

## Conclusion

The research findings indicated a notable poor knowledge about obstetric fistula among antenatal attendees at Faji Kunda and Farafenni Health Facilities. Additionally, the study revealed a significant association between knowledge about obstetric fistula and certain demographic factors such as age, educational attainment, wealth index, and occupational status. This implies that variations in age groups, levels of education, economic status (as indicated by wealth index), and occupational roles are linked to differing levels of awareness and comprehension regarding obstetric fistula among the attendees at these health facilities.

## Supporting information

S1 File SPSS Dataset (OBF). This file contains the raw data in SPSS format use for the analysis in this study.
(SAV)

S2 File Excel Dataset (OBF). This file contains the raw data in Excel format for replication of the statistical analysis.
(XLSX)

S3 File Inclusivity-in-global-research-questionnaire. This file contains the completed PLOS ONE inclusivity questionnaire.
(DOCX)

S4 File Questionnaire. This file includes the questionnaire administered to participants during data collection.
(DOCX)
