# Supplementary material for: Knowledge and associated factors of obstetric fistula among antenatal care attendees at Faji Kunda and Farafenni health facilities, The Gambia
Source: PLoS One. 2025 Sep 24;20(9):e0331130. doi: 10.1371/journal.pone.0331130 (PMC12459818; doi:10.1371/journal.pone.0331130)
Supplement: S4 File — This file includes the questionnaire administered to participants during data collection. (DOCX) [file pone.0331130.s004.docx]

**QUESTIONNAIRES**

**SECTION A: DEMOGRAPHIC DATA**

**SUBJECT’S ID.________________________**

**Instructions:** Please circle the correct provided options.

1. **Age**...............
2. **Marital status**
3. Marriage
4. Divorced
5. Widowed
6. Single
7. **Age of first pregnancy**…………..
8. **Number of pregnancies**…………..
9. **Number of children**………………
10. **Educational level**
11. No formal education
12. Primary education
13. Secondary education
14. Tertiary education
15. **Occupational status**
16. Housewife
17. Government employee
18. Private employee
19. Student
20. Farmer
21. Business
22. Others (specify)…………….
23. **Residence**
24. Rural
25. Urban
26. **Wealth index**
27. Poorest
28. poor
29. Medium
30. Rich
31. Richest
32. **Husband/partner Educational level**
33. No formal education
34. Primary education
35. Secondary education
36. Tertiary education
37. **Husband/partner occupational status**
38. Housewife
39. Government employee
40. Private employee
41. Student
42. Farmer
43. Business
44. Others (specify)…………….

**SECTION B:** **KNOWLEDGE OF OBSTETRIC FISTULA**

1. **What is an obstetric fistula?**
2. A hole between the vagina and the bladder or a hole between the vagina and rectum
3. It is when women cannot keep their urine for long
4. It is caused by evil spirits
5. Others (specify)……………………
6. **What are the common types of obstetric fistula?**
7. Vesicovaginal fistula
8. Rectovaginal fistula
9. Both
10. I do not know
11. **What are the risk factors associated with obstetric fistula? (choose all that applies)**
12. Early pregnancy
13. Home delivery
14. Female Genital Mutilation
15. Evil spirits
16. Breach of a prohibited act
17. Prolonged labor
18. Malnutrition of the mother
19. Operative delivery
20. Early marriage Others (specify…………………………………….)
21. I do not know
22. **What are the symptoms of obstetric fistula? (choose all that applies)**
23. Urinary incontinence
24. Fecal incontinence
25. Foul-smelling vaginal discharged
26. Stomach ache
27. Pain while having sex
28. Vulva irritation
29. Others (specify…………………………………….)
30. I do not know
31. **Is obstetric fistula preventable?**
32. Yes
33. No
34. **What are the preventive methods of obstetric fistula? (choose all that applies)**
35. Delaying the first age of pregnancy
36. Cessation of harmful traditional practices
37. Timely visit/seeking of skilled obstetric care
38. Eradicating poverty
39. , Empowering girls'/women's education
40. Family planning use
41. Skilled care at birth
42. Others (specify…………………………………….)
43. I do not know
44. **Is obstetric fistula treatable?**
45. Yes
46. No
47. **If yes, how can it be treated?**
48. Medically
49. Surgery
50. Traditional
51. **Can the use of contraceptives/ family planning reduce the risk of obstetric fistula?**
52. Yes
53. No
